# OTA Prevention and Detoxification by Actinobacterial Strains and Activated Carbon Fibers: Preliminary Results

**DOI:** 10.3390/toxins10040137

**Published:** 2018-03-24

**Authors:** Rhoda El Khoury, Elodie Choque, Anthony El Khoury, Selma P. Snini, Robbie Cairns, Caroline Andriantsiferana, Florence Mathieu

**Affiliations:** 1 Laboratoire de Génie Chimique, Université de Toulouse, CNRS, Toulouse, France; Avenue de l’Agrobiopole-BP 32607-Auzeville-Tolosane 31326 CASTANET-TOLOSAN CEDEX; rhoda.elkhoury@ensat.fr (R.E.K.); elodie.choque@u-picardie.fr (E.C.); anthony.khoury4@net.usj.edu.lb (A.E.K.); selma.snini@ensat.fr (S.P.S.); robbiecairns111@gmail.com (R.C.); caroline.andriantsiferana@iut-tls3.fr (C.A.); 2 Unité de Recherche Biologie des Plantes et Innovation (BIOPI-EA 3900), Université de Picardie Jules Verne, 33 rue Saint Leu, 80039 Amiens CEDEX, France

**Keywords:** ochratoxin A, biocontrol, detoxification, actinobacteria, activated carbon fibers

## Abstract

Ochratoxin A (OTA) is a mycotoxin produced by several species of *Aspergillus* and *Penicillium* that contaminate food and feed raw materials. To reduce OTA contamination, we first tested in vitro, actinobacterial strains as potential biocontrol agents and afterward, through a physical decontamination method using activated carbon fibers (ACFs). Actinobacterial strains were screened for their ability to reduce OTA in solid co-culture with *A. carbonarius*, which is the major OTA-producing species in European vineyards. Four strains showed a high affinity for removing OTA (67%–83%) with no significant effect on fungal growth (<20%). The mechanism of action was first studied by analyzing the expression of OTA cluster genes (*acOTApks*, *acOTAnrps*, *acOTAhal*) by RT-qPCR showing a drastic reduction in all genes (7–15 times). Second, the ability of these strains to degrade OTA was assessed in vitro on ISP2 solid medium supplemented with OTA (100 µg/L). Two strains reduced OTA to undetectable levels. As for the physical method, high adsorption rates were obtained for ACFs at 0.8 g/L with a 50% adsorption of OTA in red wine by AC15 and 52% in grape juice by AC20 within 24 h. These promising methods could be complementarily applied toward reducing OTA contamination in food chains, which promotes food safety and quality.

## 1. Introduction

Ochratoxin A (OTA) is a mycotoxin produced by several fungi belonging to the *Aspergillus* genus such as *A. niger*, *A. carbonarius*, and *A. westerdijkiae* as well as *Penicillium* genus such as *P. verrucosum* and *P. nordicum* [1]. These fungi are common contaminants of food and feed raw materials and, therefore, responsible for human and animal exposure to OTA. OTA has carcinogenic, nephrotoxic, immune-suppressive, and teratogenic effects. It is consequently classified by the IARC in the Group 2B as a potential carcinogen [2]. Due to its toxic effects, the European Commission (EC) has set maximum levels for OTA concentration in agricultural foodstuffs including 3 µg/kg in cereal-based products, 2 μg/kg in wine and grape juice, and 0.5 µg/kg in baby foods for infants and young children. Therefore, the EC recommends a tolerable weekly intake that does not exceed 120 ng/kg/bw [3]. OTA contamination appears to be widespread since its producers belong to two fungal genera with each requiring different conditions for growth and mycotoxin production [1]. Furthermore, climate change has highly influenced mycotoxin distribution leading to the further prevalence of OTA contamination and fluctuations in contamination levels. Moreover, over the last few years, wine has been considered the second main source of OTA in the European diet. Contamination of grapes and wine by OTA then constitutes a serious health and economic problem in southern European countries, which are the largest wine producers worldwide [4,5].

Preventive measures that rely on implementing good agricultural practices (GPA) are already carried out directly in the vineyards. This includes pest management (cochineal and other insects), early harvest, elimination of moldy grapes and bunches, and more. Although these techniques are crucial for maintaining healthy vineyards, they can be costly, time-consuming, and insufficient for eradicating the risk of mycotoxin contamination [6,7,8]. It is then necessary to develop complementary methods to reduce OTA exposure through food and feed consumption by humans and animals. Two main types of approaches could be developed including preventive methods that could be applied in the field or during storage and acting directly on fungal development and/or on mycotoxin production as well as curative methods that aim to detoxify contaminated matrices by eliminating the produced mycotoxin.

On one hand, phytosanitary products were favored as a preventive approach for limiting fungal development in the fields. However, the sanitary and environmental impacts of these products as well as the modification of the nutritional value of treated crops have led researchers to develop alternative preventive strategies. For example, natural compounds such as spices, plant extracts, and essential oils have been proven to reduce mycotoxin production by targeting their biosynthesis cluster of genes [9,10,11]. Microorganisms have also demonstrated different capacities to reduce the final mycotoxin concentration. A few biocontrol products have also been developed for limiting postharvest fungal diseases on some fruits and vegetables. As for OTA contamination in grape berries, the effect of yeasts and their different mechanisms of action were thoroughly described [12]. Also, a *Bacillus licheniformis* strain was able to inhibit the growth of *A. westerdijkiae* (34%) and to remove 92.5% of the initial OTA concentration [13]. Actinobacterial strains have as well demonstrated an ability to reduce mycotoxin concentration without affecting fungal growth [14,15,16].

On the other hand, as part of a curative approach, literature reports significant reductions in OTA concentration over time in red or sweet wine using authorized oenological fining agents such as chitosan, bentonite, chitin, egg albumin, or potassium caseinate and activated carbon powders (ACPs) where the latter proved to be the most efficient [12,17,18,19,20]. In fact, when used at different concentrations ranging from 0.05 to 0.36 g/L, ACPs are able to reduce an initial OTA concentration (1 to 5 μg/L) by 50 to 100% in 12, 24, or 48 h [19,21,22,23]. Additionally, this adsorbent could eliminate OTA at much lower doses than other additives and a dose of 0.5 g/L is industrially preconized. The major disadvantage of ACPs is that it can sometimes alter the organoleptic properties and color of wine by adsorbing molecules of interest such as polyphenols [19] and a filtration step is required to finish the operation. Activated carbon fibers (ACFs) could represent a compromise since they show a similar adsorption capability with several supplementary advantages. They are easier to remove after treatment (no filtration step) and could possibly be used in a continuous process, have a potentially higher adsorption rate, and a very high local adsorbent concentration. Moreover, one of the characteristics of ACFs is that these materials behave like molecular sieves due to a narrow pore size distribution [24,25]. Furthermore, ACFs in the form of tissues could potentially provide an advantageous alternative to ACPs since their fabric or sponge-like material could be more easily introduced in the grape juice or winemaking process than powder. This property could also be particularly interesting for wine treatment since it could theoretically reduce the risk of eliminating larger molecules that have to be preserved such as polyphenols. However, this would require further focused study to confirm. To our knowledge, this study is the first to provide results on the use of ACFs for OTA decontamination in wine.

In this context, this study provides promising results for the development of two main approaches to reduce the occurrence and concentration of OTA in food chains. On one hand and, as a preventive approach, we studied the effect of actinobacterial strains to reduce OTA production in a co-culture with *A. carbonarius* on a solid medium and their effect on OTA cluster genes. On the other hand, as a curative approach, we first evaluated the capacity of four selected actinobacterial strains to degrade OTA. Then we tested the capacity of these strains and several types and doses of ACFs for the removal of OTA via adsorption in different liquid matrices (acidified water, red grape juice, and red wine). These different approaches are complementary methods that could be applied together to improve the global OTA reduction in food chains, which promotes food safety.

## 2. Results and Discussion

### 2.1. Actinobacterial Strains for the Prevention and the Detoxification of OTA

#### 2.1.1. Screening of the Ability of Different Actinobacterial Strains to Reduce OTA Concentration

**Figure 1:**
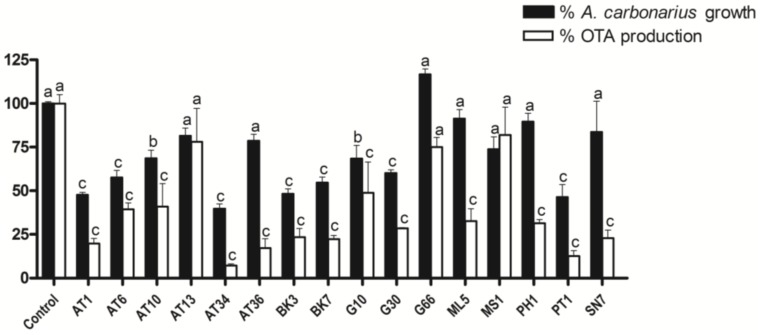
Effect of different actinobacterial strains on the growth of *A. carbonarius* and OTA’s concentration in the solid co-culture medium. Data with different letters are significantly different (One-way ANOVA, Dunnett Post-Hoc Test, *p*-value < 0.05).

The screening consisted of testing the capacity of 16 actinobacterial strains to reduce OTA’s concentration in the solid co-culture medium without affecting fungal growth. Thirteen strains presented significant OTA reduction capabilities where nine of which (AT1, AT6, AT10, AT34, BK3, BK7, G10, G30, and PT1) had also significantly decreased *A. carbonarius* mycelial area (>20%) and were subsequently discarded from the study. Four strains were finally retained, which include ML5, PH1, SN7, and AT36. This reduces OTA in solid medium by 67% to 83% (*p*-value < 0.01) with no significant reduction of fungal growth (<20%) (Figure 1).

In addition, alteration of fungal growth would lead to an imbalance in the ecological niche and promote the appearance of other microorganisms whose occurrence can be unexpected and possibly produce more toxic metabolites. The soil environment is a complex ecosystem harboring an indigenous microbiota. Although some microorganisms and especially fungal species may be viewed as harmful crop pathogens, they could also act as beneficial biocontrol agents due to hyphal growth and proliferation. Any disturbance of the soil ecosystem leading to an imbalance in the microbiota could increase the colonization of soilborne phytopathogens or even plant/root pests [26].

Only a few works have taken into account the impact of biocontrol agents on fungal growth [9,11,14,15,27]. On the contrary, recent studies on the development of biocontrol strategies to counteract mycotoxin contamination aims to prevent the growth of mycotoxigenic fungi [28,29,30,31,32]. Moreover, these studies suggest the pulverization of biocontrol agents as a treatment. However, the use of actinobacterial strains, which is proposed in this study, could also consist on their introduction into the harvest soil known as their natural habitat. The four selected strains, all belonging to the *Streptomyces* genera, are endophytic and rhizosphere bacteria that are able to colonize hostile environments, which is a fact highlighting their sustainability as phyto-protective agents [33].

#### 2.1.2. Transcriptomic Regulation of OTA Production by Actinobacteria in Solid Co-Culture with *A. carbonarius*

**Figure 2:**
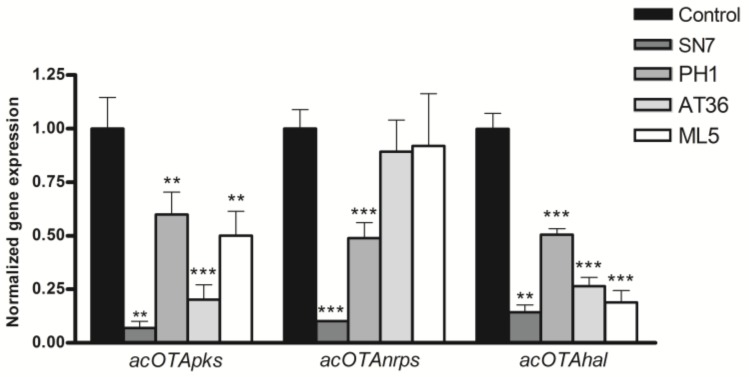
Levels of normalized gene expression of OTA cluster genes *acOTApks*, *acOTAnrps*, and *acOTAhal* in *A. carbonarius* when the latter is in co-culture with SN7, PH1, AT36, and ML5 actinobacteria strains (Two-way ANOVA, Bonferroni Post-Hoc Test, ** *p*-value < 0.01; *** *p*-value < 0.001).

Ochratoxin A is a final product of an enzymatic cascade where the involved enzymes are encoded by a number of genes clustered in the same chromosomic region. Unlike other well-known mycotoxins, OTA’s gene cluster is partially elucidated and only three genes called *acOTAhal*, *acOTAnrps*, and *acOTApks* are coding, respectively, for a halogenase, which is a non-ribosomal peptide synthase and a polyketide synthase characterized to this date [34,35]. The expression of these genes was analyzed in *A. carbonarius* in solid co-culture with SN7, PH1, AT36, and ML5 strains in order to determine whether the observed OTA reduction is the result of an inhibition of OTA synthesis at the transcriptomic level. Gene expression of *acOTApks* and *acOTAhal* were significantly reduced by all strains. As for *acOTAnrps*, its expression was only reduced by SN7 and PH1 strains. In fact, gene expression reductions were the most drastic in the presence of the SN7 strain that reaches 85.6%, 89.8%, and 93.1% for *acOTAhal*, *acOTAnrps*, and *acOTApks*, respectively (see Figure 2). Therefore, soil-ubiquitous bacterial strains could be used as biological antagonists on several *Aspergillus* species and other mycotoxigenic genera [36,37]. These globally distributed bacteria mainly belong to *Bacillus*, *Streptomyces*, *Pseudomonas*, and *Agrobacterium* genera. They are able to produce endospores and wide-range bioactive compounds acting to prevent mycotoxin production, which encourages efforts for their use as potential efficient biocontrol agents [33,36]. Another study has also focused on the ability of *Saccharomyces cerevisiae* to reduce OTA’s production by decreasing the expression of the *pks* gene in two ochratoxigenic strains of *A. carbonarius* and *A. ochraceus* [38].

In the present study (concerned only with the transcriptomic analysis), the reduction of OTA’s concentration by the SN7 *Streptomyces* strain is another example where the production regulation can occur at a transcriptomic level. Similar transcriptomic mechanisms of *Streptomyces* spp. and *Bacillus* spp. were already described. The mechanisms are described mainly for aflatoxins where co-cultures as well as bacterial metabolites such as blasticidin and its derivatives, aflastatin A and dioctatin A, presented an anti-aflatoxinogenic effect by downregulating the expression of aflatoxin cluster genes [16,36,39,40,41,42,43].

#### 2.1.3. OTA Degradation and Adsorption Assays

**Figure 3:**
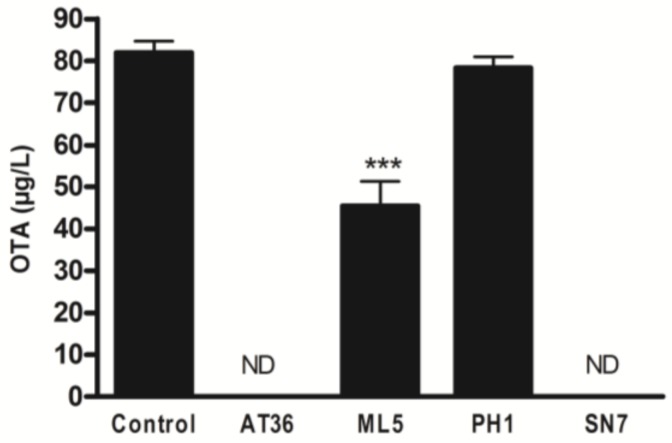
Residual OTA concentration (µg/L) in ISP2 medium supplemented at 100 µg/L with OTA following a five-day incubation period at 28 °C of four actinobacterial strains (AT36, ML5, PH1, and SN7). ND = not detectable (One-way ANOVA, Dunnett Post-Hoc Test, *** *p*-value < 0.001).

In order to evaluate the ability of actinobacterial strains to reduce OTA concentration after its production, biodegradation, and adsorption assays were conducted. Among the four selected strains, the culture of SN7 and AT36 strains on OTA-supplemented ISP2 agar, led to undetectable levels of the analyzed toxin. A lesser reduction was observed with the ML5 strain where 55 ± 7.1% of the initial OTA concentration was retrieved (see Figure 3). However, no reduction was observed with the PH1 strain. These results highlight the different mechanisms of action that could be adopted by these strains including OTA degradation. Biodegradation assays by lactic acid bacteria and yeast are widely described in literature with varying efficiencies going up to 94% of 50 µg/L of OTA [44]. In addition, there are two main types of OTA biodegradation pathways including the hydrolysis of the amide bond that links the L-β-phenylalanine molecule to the OTα moiety and the hydrolysis of the lactone ring leading to an opened lactone form of OTA [45]. Therefore, the HPLC/DAD/FLD analysis of the medium extract in presence of SN7 revealed the presence of an unidentified component correlated with the disappearance of the OTA peak.

This study also aimed to evaluate adsorption of OTA on bacterial spores for the resistance and the handling facility of these latter, which could present a significant advantage in terms of developing a practical biocontrol agent [46]. Nonetheless, none of the four tested strains were able to bind OTA on their spores (see Table 1). However, many studies have proved the capacity of lactic acid bacteria and yeast cell walls to bind mycotoxins including OTA [47,48,49,50]. This mechanism was linked to the modification of the cell membrane structure (peptidoglycan, polysaccharides, and glycoprotein) following a heat or acid treatment [48].

### 2.2. Removal of OTA via Adsorption on Activated Carbon Fibers (ACFs)

#### 2.2.1. Textural Characterization of ACFs

**Figure 4:**
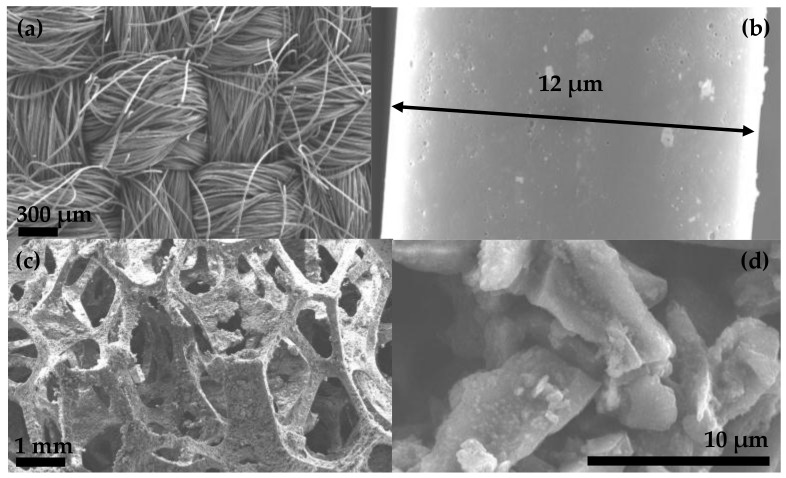
Scanning Electron Microscopy (SEM) images of (**a**) D1200 woven fabric; (**b**) D1200 fiber; (**c**) D800 foam structure; and (**d**) D800 deposited activated carbon.

Different ACFs were used in this study including AC10, AC15, AC20, and D1200 whose fibers diameters vary between 10 and 16 µm, which is shown in Figure 4a,b. The D800 fabric is a foam made of polyurethane with activated carbon powder impregnation (see Figure 4c). As shown in Figure 4d, the size of the deposited activated carbon grain does not exceed 15 nm. Moreover, Table 2 summarizes several physical characteristics of the different ACFs. Woven ACF samples contained binodal pores with tight pore size distributions in the domain of micropores. For AC20, two pore size distributions were observed including one between 0.5 nm and 0.8 nm and the other between 0.8 nm and 2.5 nm. Smaller size pore diameters were obtained for AC10: 0.4 nm and 0.8 nm and 0.8 nm and 1.5 nm. The first pore size distribution seems too narrow to allow the penetration of OTA in the pores. Conversely, for the second pore size distribution, an important part was slightly larger than the size of OTA (except for AC10). Penetration of molecules in the pores depends on their spatial orientation. OTA may then penetrate into a part of the micropores but with some difficulty. Finally, even if OTA may penetrate into these micropores, an unfavorable orientation of this molecule may block a part of the micropores and decrease adsorption capacity. Conforming to the technical characteristics provided by the manufacturer, all woven ACFs present high values of specific surface area and microporous volumes (around 90% of total volume). The samples of AC15, AC20, and D1200 exhibited Brunauer–Emmett–Teller (BET) surface areas of approximately 1.9, 2.2, and 1.5 times more than AC10 samples, respectively. These adsorbants, in particular AC20, are expected to have high adsorption capacities due to the higher pore diameters and their larger BET surface area. D800 foam was mainly mesoporous (86% of total volume) and possesses very low BET surface area (40 m^2^/g) while the activated carbon used for the deposition has a BET surface area of 800 m^2^/g (supplier data). This significant difference between measured BET surfaces may be explained by an important amount of non-porous polyurethane in comparison with the quantity of the deposited activated carbon.

#### 2.2.2. OTA Adsorption Depends on ACFs

**Figure 5:**
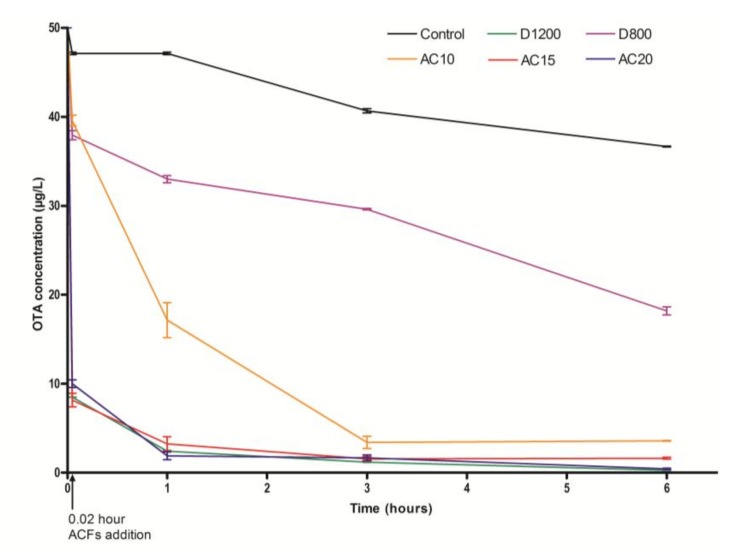
OTA adsorption kinetics in AWM with the different ACFs (D1200, D800, AC10, AC15, and AC20) at 0.8 g/L. ACFs were added at 0.02 h (1 minute). All data presented statistically significant differences (*p*-value < 0.001) when compared to the control (Two-Way ANOVA, Bonferroni Post-Hoc Test).

Adsorption assays were conducted in acidified water medium (AWM) for all ACFs. Different adsorption rate trends could be observed in Figure 5. In addition, adsorption rates were higher for AC15, AC20, and D1200 rather than for AC10 and D800. More than 80% of OTA is eliminated as soon as ACFs were vortexed in the AWM, which highlights the high affinity between these ACFs and OTA. In such acidic conditions (pH 3.4), OTA is uncharged. The adsorption mechanism is then mainly due to electron-donor interaction between π-electrons of aromatic molecules and π-electrons on ACFs surface. These high adsorption rates were due to the large external surface areas in which the small size of the fibers and the availability of micropores directly on the surface of the fibers limit mass transfer resistance inside the pores. This behavior is different from usual ACPs characterized by dispersed pore size distribution in which the presence of mesopores increases internal mass transfer resistance [51,52].

AC20, AC15, and D1200 exhibited high adsorption capacities since nearly all of the OTA was eliminated. This is in part due to their large pore diameters which allows easy access for OTA (OTA topological polar surface area: 1.13 nm^2^). Even though access to certain pores was limited in this low concentration range, there were enough pores and certainly sufficient external surface area to adsorb all the molecules. The lower adsorption rate for AC10 could be due to its lower pore size distribution between 0.8 nm and 1.5 nm. Additionally, the molecule size is of the same magnitude, which makes penetration more difficult. Finally, for the D800 foam (polyurethane and activated carbon), the lower adsorption rate could be due to the limited accessibility to the activated carbon inside the structure of the foam compared to other ACFs. Therefore, the diffusivity inside the material and through the liquid inside of the foam could influence mass transfer. High adsorption capacities could be explained by the mesoporous nature of the deposited activated carbon. In addition, even if the quantity of activated carbon is small, at low concentration, there are enough sites available to adsorb OTA.

#### 2.2.3. Effect of ACFs Concentration on OTA Adsorption in AWM

**Figure 6:**
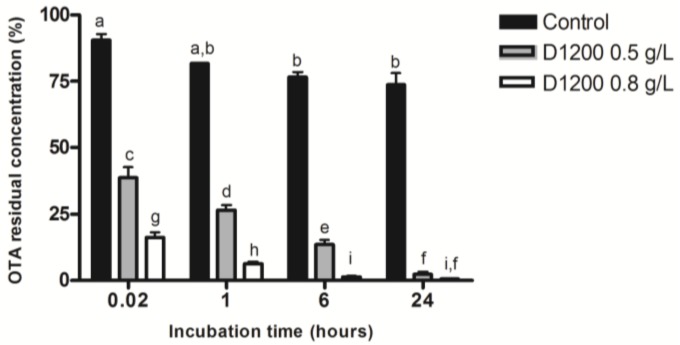
OTA residual concentration (in % ± SEM) in Acidified Water Medium (AWM) after addition of different concentrations D1200 ACF (OTA initial concentration—50 µg/L). Data different letters are significantly different (Two-way ANOVA, Bonferroni Post-Hoc Test, *p*-value < 0.05).

D1200 was selected for this study since it is one of the woven ACFs that gave the highest performances in the previous experiments. Results show that more than 50% of OTA is adsorbed as soon as ACFs were vortexed in the matrix (0.02 h) regardless of their concentration. High OTA removal rates were observed after the first hour. For example, an ACFs concentration of 0.8 g/L allows an OTA removal of 92% after one hour. After 24 h, the residual OTA concentration is less than 3% with both D1200 concentrations (see Figure 6). The performance achieved with ACFs is better than most authorized additives. Actually, equivalent removal rates are reported for chitosan concentrations between 5 and 40 g/L [53] or bentonite at a concentration of 40 g/L [54]. Nonetheless, compared to ACPs, ACFs efficiency are equivalent to the one obtained by Gambuti et al., 2005, for different types of ACPs at a concentration of 0.5 g/L, which showed OTA removal rates between 68% and 98% [55].

#### 2.2.4. Effect of OTA Concentration on Its Adsorption by ACFs in AWM

Results presented in Table 3 show that for each OTA concentration and treatment time, the three types of ACFs gave similar OTA removal rates. An immediate OTA adsorption from 60% to 88% was observed immediately after the addition and vortex of the ACFs (0.02 hour). After 1 hour, OTA adsorption was improved by whatever the ACFs used. For example, this reached 96% of an initial OTA concentration of 50 µg/L with AC20. Surprisingly, after 6 hours of ACFs incubation, the worst performances are obtained for the lowest OTA concentration (2 µg/L). Generally, the time required to reach an adsorption equilibrium depends on the initial concentration. However, this behavior can be inverted from one molecule to another. The equilibrium time can be higher at a low concentration as reported by Al Mardini et al. (2009) for Bromacil adsorption on ACPs [56]. The authors obtained equilibrium times of 2 hours and 6 hours for Bromacil concentration of 5 µg/L and 500 µg/L, respectively. Conversely, Fallou et al. (2016) reported a contact time of 50 hours with ACFs to reach an equilibrium for low concentrations (10 µg/L of diclofenac) and 10 days for an initial concentration of 10 mg/L [57]. For OTA, comparable removal rates could then be expected at low concentration, provided that the contact time was higher.

#### 2.2.5. OTA Adsorption by ACFs in Red Grape Juice and Red Wine

**Figure 7:**
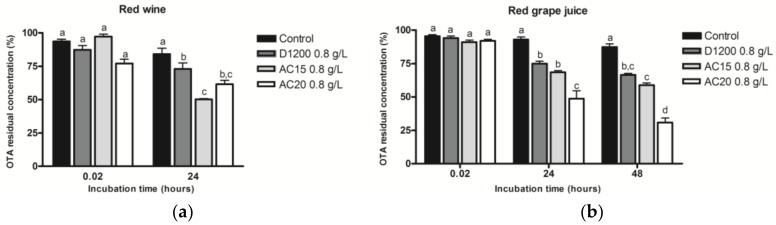
OTA residual concentration (in % ± SEM) in **(a)** red wine and **(b)** red grape juice after addition of 0.8 g/L ACFs. OTA initial concentration is of 50 µg/L in red wine and 100 µg/L in red grape juice. Data with different letters are significantly different (Two-way ANOVA, Bonferroni Post-Hoc Test, *p*-value < 0.05).

OTA adsorption by ACFs was also evaluated in red wine and red grape juice matrices. A higher initial OTA concentration (100 µg/L) was used for this test in red grape juice since heat treatment applied to the latter was not sufficient for the destruction of fungal spores, especially since the storage period could last for one year. Results presented in Figure 7 clearly show that OTA’s removal in complex matrices at an equivalent acidic pH (pH 4.1 for red wine mixture and pH 3.7 for red grape juice) occurs at a lesser intensity and slower kinetics than in AWM. In fact, after a 1 minute (0.02 hour) incubation time with any of the ACFs, no significant differences could be observed in OTA’s residual concentrations when compared with the control. Following 24 h of incubation, all ACFs presented significant adsorption capabilities with different efficiencies in red wine and grape juice. With these results, it can be said that AC15 and AC20 are the most efficient in adsorbing OTA by reaching 39% to 50% in red wine and 32% to 48% in red grape juice. In both matrices, D1200 presents the lowest adsorption potency of 27% in red wine and 25% in red grape juice. After 48 hours, in red grape juice, AC20 presented the best OTA removal rate of 70%.

Reduced efficiencies (adsorption capacities and rates) of the ACFs in red wine and grape juice could be due to adsorption competition between OTA and other molecules present in those real matrices. These molecules compete with OTA by two different mechanisms, which include smaller molecules (smaller sizes and comparable molecules) competing directly with OTA on adsorption sites within the micropores and larger molecules can be adsorbed on mesopores or on ACFs surface, which would block OTA’s entry to micropores [52,58]. Conversely, regardless of OTA concentration, when OTA is the only molecule present in AWM, ACFs adsorption sites are more accessible and the available surface is high enough to allow the adsorption of all OTA molecules without any competition effect. However, in a multi-constituent and highly concentrated mixture, local adsorption sites are preferentially occupied by other molecules, which reduce their availability for OTA. This type of behavior was also previously observed in the case of ACPs adsorption of two phenolic compounds. The individual adsorption rates of phenol and parahydroxybenzoic acid (PHBA) are of the same order of intensity. However, in an equimolar mixture, the adsorption rate of PHBA is twice that of phenol. Moreover, the experiment showed an increase of the preferential adsorption of PHBA in the midst of decreasing phenol concentration. In a complex matrix, chemical properties of molecules such as solubility, hydrophobicity, and pKa greatly influence adsorption capacities [59].

The lesser efficiency of D1200 could be explained first by its physical characteristics. The S_BET_ surface area is smaller and its porous volume corresponds to almost half of the other two tested ACFs. Consequently, fewer sites are then available. This suggests the occurrence of stronger competition effects, which results in lesser OTA adsorption. Second, the difference in efficiencies could also be linked to the chemical nature of the D1200 ACF. Generally, the strength of the interactions between molecules and activated carbons depend on both the surface chemistry of the activated carbon (lactonic, phenolic, carboxylic, and basic groups) as well as on the chemical properties of the putative adsorbed molecules. Therefore, the activated carbon affinity for the targeted molecules will depend on their chemical properties [60]. In fact, both the precursor and the activation method used in the fabrication process of D1200 are different from those for AC15 and AC20. Consequently, the chemical surface groups are not the same, which could explain the different affinity for OTA and the possibility of preferential adsorption of other molecules present in the matrix.

## 3. Conclusions

In this study, we provide promising results for developing alternative strategies to counteract OTA contamination in food chains. Actinobacterial strains could be used as preventive agents by introducing them in harvest soil to compete with *A. carbonarius*, which leads to inhibition of OTA production at the transcriptomic level. Such microorganisms could also be considered detoxifying agents in a curative approach due to their ability to degrade OTA. Detoxification of OTA contaminated matrices could also be potentially achieved by the use of ACFs in liquid matrices. However, their effects on other wine substances (i.e. phenolics or other) must be further studied. This study is the first to provide data on their capacity to decrease OTA levels by an adsorption phenomenon. The two described methods could then subsequently be used to reduce the risk of OTA contamination starting from the field to the end product.

## 4. Materials and Methods 

### 4.1. Chemicals

The Ochratoxin A (pKa = 7.1) standard was purchased from Sigma-Aldrich (Saint Quentin Fallavier, France). All analytical grade solvents used in the extraction and high-performance liquid chromatography (HPLC) analysis were purchased from Thermo Fisher Scientific (Illkirch, France). Ultrapure water used for HPLC and for molecular biology procedures was purified at 0.22 µm by an ELGA purification system (ELGA LabWater, High Wycombe, United Kingdom).

### 4.2. Strains, Media, and Culture Conditions

Sixteen actinobacterial strains (AT1, AT6, AT10, AT13, AT34, AT36, BK3, BK7, G10, G30, G66, ML5, MS1, PH1, PT1, and SN7), previously isolated from Algerian soils were used in this study. Strain revivification was performed on liquid ISP2 medium (10 g Malt Extract, 4 g Yeast Extract, 4 g d-glucose per 1 L) and incubation was held in an orbital shaker set at 28 °C for 10 days. Pre-cultures were then performed on solid ISP2 medium and incubated at 28 °C for five days. *Aspergillus carbonarius* S402 was previously isolated from a Lebanese vineyard and pre-culture was harvested on Czapeck Yeast Extract Agar (CYA) medium (Sucrose 30 g, Yeast Extract 5 g, NaNO_3_ 2 g, K_2_HPO_4_ 1 g, KCl 0.5 g, MgSO_4_ 0.5 g, FeSO_4_ 0.01 g, ZnSO_4_.7H_2_O 0.1g, CuSO_4_.5H_2_O 0.005 g, per 1 L).

### 4.3. Co-Culture of Actinobacterial Strains and A. Carbonarius

**Figure 8:**
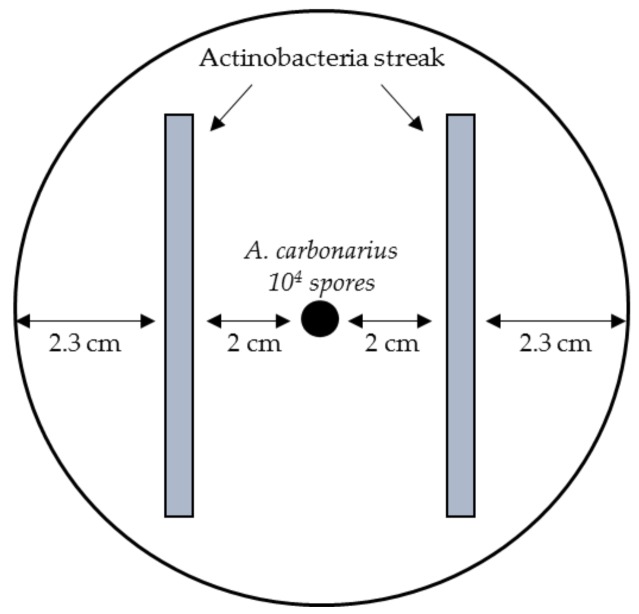
Schematic representation of actinobacteria and *A. carbonarius* co-culture inoculation on solid ISP2 medium.

Bacteria, from five-day old solid pre-culture, were inoculated as streaks at 2 cm from the center of the Petri dish on ISP2 medium (10 g Malt Extract, 4 g Yeast Extract, 4 g d-glucose, and 15 g agar per 1 L). Ten microliters of an *A. carbonarius* spore solution at 10^6^ spores/mL in 0.05% Tween 80 were centrally inoculated, which is shown in Figure 8. Cultures were incubated at 28 °C for five days. At the end of the incubation period, fungal growth was assessed by calculating the mycelium area. The entire medium was then extracted using 25 mL of acidified chloroform (0.2% *v*/*v* of acetic acid) and left to macerate while shaking overnight. Extracts were then filtered through 1WPS Whatman filters and evaporated to dryness under a Rotavapor at 60 °C. Dry extracts were re-suspended with 2 mL of methanol and purified with Ochraprep Immunoaffinity columns (R-Biopharm, Glasgow, UK) following the manufacturer’s instructions before being subjected to HPLC/FLD/DAD analysis for OTA quantification.

### 4.4. OTA Degradation Assay by Actinobacteria

Bacteria were inoculated on ISP2 medium supplemented with OTA at 100 µg/L using a streak from a five-day old solid pre-culture. Cultures were incubated for five days at 28 °C with four replicates per condition. Ochratoxin A was extracted as previously described. Dry extracts were re-suspended with 2 mL of a mixture of methanol/water (50/50, *v*/*v*) and analyzed by HPLC/FLD/DAD for OTA quantification.

### 4.5. OTA Adsorption Assay on Actinobacteria Spores

Spores were collected in Tween 80 (0.05%) from a seven-day bacterial pre-culture and counted on Malassez cell. 10^6^ spores were incubated for one hour at room temperature in an OTA solution of 100 µg/L with a final volume of 1 mL. The solution was then filtered through a PVDF 0.45 µm membrane (GE Healthcare Lifesciences). The filter was further washed with 1 mL of purified water and then with 1 mL of acidified methanol (2% acetic acid, *v*/*v*). The filtrate as well as the volumes of both washes were filtered through a 0.45 µm PVDF membrane before being analyzed by HPLC/FLD/DAD for OTA quantification.

### 4.6. Analysis of Relative Expression of the OTA Cluster Genes by Qrt-PCR

Transcriptomic analysis was only carried out on the SN7 strain. Co-culture of *A. carbonarius* and SN7 actinobacterial strain was performed as described above with minor changes. Before inoculation of strains on the ISP2 Petri dish, the medium was layered with 8.5 cm diameter cellophane disks (Hutchinson, Chalette-sur-Loing, France) to allow the separation of mycelium from the culture medium. Co-cultures were incubated for four days at 28 °C. At the end of the incubation period, the mycelium was carefully cut from the rest of the cellophane film and finely grinded with liquid nitrogen. A maximum of 100 mg were used for total RNA purification through a RNeasy Plus Minikit (Qiagen, Hilden, Germany) that includes an on-column genomic DNA clean-up, which all followed the manufacturer’s instructions. RNA integrity and purity were checked using the Experion RNA analysis kit and software (version 3.20, 2015, BioRad, Marnes-la-Coquette, France). Synthesis of the single strand cDNA was performed on 1 μg of total RNA using the Advantage RT-for-PCR kit (Clontech, Mountain View, CA, USA) with an oligo-dT following the manufacturer’s protocol. As for the qRT-PCR analysis, the expression of targeted genes (*acOTApks*, *acOTAnrps*, *acOTAhal*) was evaluated with CFX96 Touch Real Time PCR detection system BioRad (version 3.0, 2012, Hercules, CA, USA), using SsoAdvanced Universal Sybr Green Supermix (Bio-Rad, Marnes-la-Coquette, France). Expression levels were normalized with the housekeeping gene *calmodulin*.

### 4.7. Activated Carbon Fibers (ACFs)

The activated carbon fibers ACF 10, ACF 15, and ACF 20 were supplied by KYNOL (Ozaka, Japan). The activated carbon fabric D1200 and the activated carbon impregnated foam D800 were supplied by DACARB (Asnières sur Seine, France).

### 4.8. ACFs Characterization

Textural characterization of the ACFs was obtained via nitrogen adsorption using a Micrometrics ST-2000 automated apparatus (77 K). Specific surface areas S_BET_ were calculated from the Brunauer–Emmett–Teller (BET) plot in the relative pressure range (p/p0) from 0.01 to 0.20 [61]. In order to assess micropore and mesopore volumes, Horváth-Kawazoe (HK) [62] and Barrett-Joyner-Halenda (BJH) [63] methods have been used. Pore diameter means were deduced from the total porous volume at p/p0 = 0.98 and BET surface areas. Surface structures of ACFs were observed using Scanning Electron Microscopy (SEM) (LEO 435 VP).

### 4.9. Liquid Matrices for OTA Adsorption by Acfs

Acidified Water Medium (AWM) is composed of 18.2 MΩ/cm molecular water (MilliQ, Merck KGaA, Darmstadt, Germany) adjusted at a pH of 3.4 using 10 M HCl. Grape juice was purchased from a local supermarket in Toulouse (France). Wine mixture used to test removal of OTA via adsorption on ACFs was composed of an equivalent amount of three different red wines, which all originated from the south-western region of France including Chateau Baudare (Fronton, 2013), Chateau Baudare “Perle Noire” (Fronton, 2012), and Chateau de Crouseilles (Madiran, 2012).

### 4.10. OTA Adsorption on Acfs in AWM, Red Grape Juice, and Red Wine

For all adsorption assays, the same methodology was adopted. Matrices were supplemented with adequate OTA concentration and were vortexed for 10 s and, then, agitated at 125 rpm for 1 h on a horizontal shaker. A defined amount of the chosen ACFs were then added to the matrices after 0.02 h. The matrices were then immediately returned to agitation for the duration of their allotted time. No ACFs were added in control conditions. All samples were vortexed for 10 s before withdrawal of a volume of 1 mL of each. Collected samples were filtered into a tainted vial on a 0.45 μm PVDF membrane and stored at −20 °C until HPLC analysis.

### 4.11. OTA Quantification by HPLC/FLD/DAD

OTA was analyzed by C18 Spherisorb ODS2 column, 150 × 4.6 mm, 5 µm, 120 Å (Waters, Saint-Quentin, France) using a Dionex Ultimate 3000 UHPLC (Thermo Scientific, France) apparatus. An isochratic flow was delivered at 1 mL/min containing 49% acidified water (0.2% acetic acid, *v*/*v*) and 51% acetonitrile. The injection volume was 100 µL. OTA was detected using an FLD detector with 332/466 nm excitation/emission wavelengths and spectrum was further confirmed by a diode array detector (DAD) coupled to the apparatus. OTA quantification was calculated according to a standard calibration curve with concentrations ranging between 20 and 500 µg/L.

### 4.12. Statistical Analysis

Differences between the varying actinobacterial strains and control cultures were analyzed using a One-Way Analysis of Variance (ANOVA) followed by a Dunnett Post-Hoc Test. Statistical analysis for OTA absorption on ACFs were analyzed using Two-Way ANOVA (*p*-value < 0.05) followed by a Bonferroni Post-Hoc Test. Data analysis was carried out with GraphPad Prism 4 software (GraphPad Software, La Jolla, CA, USA). Differences were considered to be statistically significant when the *p*-value was lower than 0.05.

## Figures and Tables

**Table 1:** Residual OTA concentration (µg/L) in an aqueous solution with an initial OTA concentration of 100 µg/L following a one-hour incubation with 10^6^ spores/mL of actinobacteria (One-way ANOVA, Dunnett Post-Hoc Test, *p*-value > 0.05).

Strain	OTA µg/L ± SEM			OTA Recovery (%)
	Filtrate	Water wash	Methanol wash	
Control	97.1 ± 3	0.8 ± 0.4	3.5 ± 0.1	101.4 ± 3.8
AT36	105.7 ±2	2.1 ± 0.6	1.6 ± 0.3	109.4 ± 2.9
ML5	111.3 ± 5	0.4 ± 0.4	0.1 ± 0.1	111.8 ± 5.7
PH1	106.6 ± 1	1 ± 0.7	0.4 ± 0.3	108 ± 1.5
SN7	107.4 ± 1	1.7 ± 0.5	0.2 ± 0.2	109.3 ± 2.2

**Table 2:** Physical characteristics of ACFs.

Sample	AC10	AC15	AC20	D1200	D800
Supplier	KYNOL			DACARB	
Reference	ACC-5092-10	ACC-5092-15	ACC-5092-20	TIS-KIP-1200	MOU-CS-800
Precursor	Novoïd fiber			Phenolic resin	Polyurethane foam impregnated with activated carbon
BET surface area (m^2^/g)	940	1758	2032	1428	40
Mesoporous volume (cm^3^/g)	0.037	0.074	0.105	0.050	0.049
Microporous volume (cm^3^/g)	0.358 (90% volume)	0.681 (90% volume)	0.780 (88% volume)	0.542 (92% volume)	0.008 (14% volume)
Size pore diameter distribution (nm)	0.4–0.8 0.8–1.5	0.5–0.8 0.8–2.2	0.5–0.8 0.8–2.5	0.5–0.8 0.8–2	-

**Table 3:** OTA residual concentration (in % ± SEM) in Acidified Water Medium (AWM) after addition of 0.8 g/L ACFs depending on OTA concentration. N.D. none determined. Statistical differences were observed for the factor “Incubation time” and “Initial OTA concentration” but not for “ACFs type” (Two-way ANOVA, Tukey Post-Hoc Test *p*-value < 0.05). No interaction was observed between factors and values are statistically different depending on incubation time or initial OTA concentration.

Initial OTA Concentration	ACFs Type	OTA Residual Concentration (%)		
		Incubation Time		
		0.02 h	1 h	6 h
2 μg/L	D1200	39.9 ± 1.3	19.0 ± 0.4	18.3 ± 1.1
	AC15	36.4 ± 1.3	24.8 ± 1.0	24.2 ± 1.2
	AC20	31.4 ± 1.1	22.7 ± 0.5	18.5 ± 0.7
50 μg/L	D1200	17.0 ± 0.1	4.8 ± 0.2	0.6 ± 0.07
	AC15	16.3 ± 0.61	6.5 ± 0.6	3.3 ± 0.23
	AC20	20.0 ± 0.5	3.8 ± 0.5	0.9 ± 0.2
200 μg/L	D1200	12.0 ± 0.38	7.4 ± 0.3	2.2 ± 0.4
	AC15	22.7 ± 0.1	12.4 ± 0.3	3.6 ± 0.7
	AC20	18.0 ± 0.3	15.2 ± 0.6	14.6 ± 0.5
